# MALDI-TOF MS Versus VITEK^®^2: Comparison of Systems for the Identification of Microorganisms Responsible for Bacteremia

**DOI:** 10.1007/s00284-016-1121-x

**Published:** 2016-09-12

**Authors:** Filomena Febbraro, Donatella Maria Rodio, Gianluca Puggioni, Guido Antonelli, Valeria Pietropaolo, Maria Trancassini

**Affiliations:** 1 Department of Pediatrics, “Sapienza” University Rome, Rome, Italy; 2 Department of Public Health and Infectious Diseases, “Sapienza” University Rome, P.le Aldo Moro, 5, 00185 Rome, Italy; 3 Department of Clinical Medicine, “Sapienza” University Rome, Rome, Italy; 4 Department of Molecular Medicine and Pasteur Institute-Cenci Bolognetti Foundation, “Sapienza” University Rome, Rome, Italy

## Abstract

We evaluated the reliability and accuracy of the combined use of MALDI-TOF MS and classical ID VITEK_2_ to identify monomicrobial infection in blood culture bottles. In total, 70 consecutive positive blood cultures were included in this study. Positive blood culture bottles were subjected to Gram staining and subcultured on solid media. Isolates grown from such culture media were used for classical ID using VITEK_2_ system. In parallel, an aliquot was subjected to a lysing-centrifugation method and used for the identification with the MALDI-TOF system. Results evidenced the correct genus and species identification of 91.4 % of microorganisms responsible for bacteremia with an agreement to the species and the genus level. If compared with the standard method VITEK_2_, our simple and cost-effective sample preparation method would be very useful for rapid identification of microorganisms using blood culture bottles. In fact, the direct method showed rapid and reliable results, especially for the gram-negative group.

## Introduction

The presence of microorganisms in the blood or bloodstream infections (BSI), confirmed by a positive blood culture [6], is a significant clinical event [24]. BSI may be hospital-acquired, community-acquired, or healthcare-associated [15]. The hospital-acquired BSI are influenced by a wide range of interventions, such as central line catheterization, which expose patients to infection; therefore nosocomial BSI are very critical in intensive care unit patients.

To date, BSI is still a major cause of mortality and morbidity [1, 18] and it is a medical emergency. Patients’ outcomes are improved by rapid identification of the responsible microorganisms since the timely initiation of antimicrobial therapy is crucial for patients’ prognosis and it is associated with higher survival rates and decreased healthcare costs.

In a large retrospective study of 5715 patients with septic shock, Kumar et al. [12] found that the survival rate decreased fivefold with inappropriate initial therapy and it was shown to be the single most powerful risk factor associated with mortality.

The standard protocol to diagnose a bloodstream infection involves liquid medium blood cultures that remain the gold standard to establish its etiology [26].

Currently, steps in routine phenotypic identification involve microscopic observation with or without gram staining, subculturing, and analysis of various biochemical reactions, performed after blood cultures positivization in order to establish the correct empiric treatment [3]. This process could take several days, especially if fastidious and slow-growing microorganisms are present [2]. As an alternative to the phenotypic identification, it is possible to perform a genotypic sequencing technology, such as 16S rRNA, but it is still expensive for a routinely diagnosis [4].

Matrix-assisted laser desorption/ionization time-of-flight mass spectrometry (MALDI-TOF MS) has been recently introduced in clinical microbiology laboratories for the identification of various microorganisms [5]. MALDI-TOF system is a soft ionization technique that allows ionization and vaporization of large nonvolatile biomolecules such as intact proteins [8]. The proteins ionized and generated during the MALDI process, are structural proteins, DNA- or RNA-binding proteins and ribosome modulation factors that produce spectral fingerprints that vary between microorganisms and that have peaks specific to genus, species, and subspecies.

Although it identifies bacteria and yeasts within a few minutes, it requires isolated colonies, which are taken from 18 to 48 h of incubation [9]. To reduce the identification timing, some investigators tried to directly inoculate the infective microorganisms from the positive blood culture bottles into the MALDI-TOF MS system [11, 23, 25]. However, direct identification requires sample preparation steps since blood culture bottles contain proteins/debris which could interfere with the spectra of the microorganisms [14]. Therefore, lysis solutions [20], the lysis-filtration method [16], or commercial kits [9] were utilized for sample preparation. However, some of these procedures are laborious and/or expensive [19]. In this study, we explored a simpler, faster, and more reliable protocol using an extraction method for the direct identification of bacteria from positive blood culture bottles in comparison to conventional method.

## Materials and Methods

### Samples

This research was performed at the Laboratory of Microbiology (DLC01) of “Umberto I” Hospital in Rome. A total of 70 consecutive positive blood cultures, obtained from patients of the Department of “Anesthesiology and Intensive Care” and “Internal Medicine” of the same hospital in the period from April to September 2015, were included in this study.

28 blood samples were collected and inoculated in BD BACTEC™ Plus Aerobic/F culture vials and incubated in the automated system BD BACTEC™ FX, whereas 42 samples were collected and inoculated in Oxoid Signal Blood Culture System Medium (OXOID S.p.A.). Only monomicrobial cultures were selected.

Blood culture bottles either signaled as positive in BD BACTEC™ FX or visualized for CO_2_ production in the Oxoid Signal Blood Culture System Medium were removed, aliquoted, and utilized for gram staining and subculture on solid media.

Isolates grown from such culture media were used for classical ID using VITEK_2_ system (bioMérieux, Inc. France) representative of our routine protocol [16].

In parallel, an aliquot taken from positive blood culture bottles was subjected to a lysing-centrifugation method (LCM) and used for the identification by the MALDI-TOF system (Bruker Daltonics, Inc.) [5].

### Technical Description of MALDI-TOF System

For microorganisms identification, protein mass patterns are compared within few minutes with commercially available reference databases (Bruker Daltonics for Microflex LT spectrometer), which include species-specific fingerprints of several bacterial and yeast isolates. Through a pattern matching procedure, mass peaks in the experimental spectra are matched with reference spectra included in the database; through this comparison, a numerical value is generated that allows accurate and rapid identification of the microorganisms to species level when score values obtained are in the range of the threshold values defined by the instrument manufacturer (score values higher than 2.0 for Bruker Biotyper software). A value higher than 2.0 is generally related to a valid identification to species level; a value between 2.0 and 1.7 is reliable identification to genus level. The identification process is performed in real-time, as soon as the sample is analyzed by the spectrometer [22].

### LCM for Positive Blood Cultures Detected by Oxoid Signal Blood system

5 ml of blood culture was centrifuged in sterile tubes at 3000 rpm for 10 min (Labofuge 200, Heraeus Sepatech). In order to obtain a bacterial pellet without red blood cells, subsequent washings with 4 ml of distilled water were carried out.

### LCM for Positive Blood Cultures Detected by BD BACTEC™ FX

5 ml of blood culture sample was centrifuged in sterile tubes at 3000 rpm for 10 min. In order to obtain a bacterial pellet without traces of blood, four washings with 4 % of sodium citrate were carried out. Finally the pellet was resuspended in 5 ml of distilled water to remove traces of sodium citrate and protein residues. The number of washings performed in order to obtain a clean pellet depended on the amount of blood present in the processed sample.

### MALDI-TOF System Identification

**Fig. 1:**
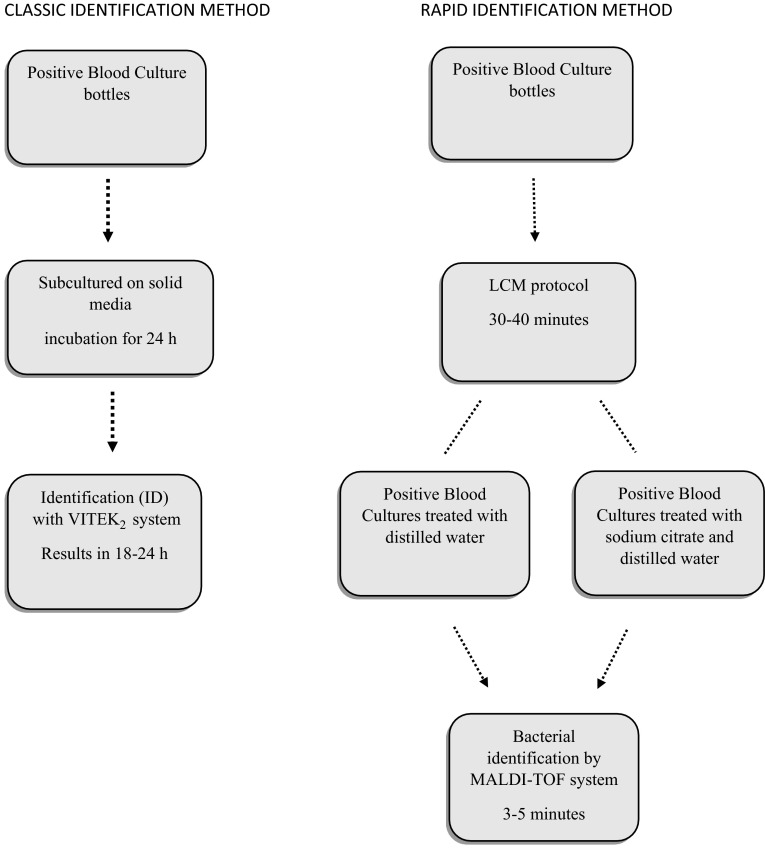
Flow chart for identification of microorganisms obtained directly from positive blood culture bottles

The pellet was smeared on a MALDI-TOF target plate and processed for a rapid microbial identification. Our protocol was set up for a blind identification of microbial species by providing the following step: a small amount of the sample was spread on two spots of a target plate. In the first spot, 1 µl of 70 % of formic acid and subsequently 1 µl of the matrix solution (acid α-cyano-hydroxycinnamic) were added, whereas in the second spot only the matrix solution was put in. The plate was placed in the mass spectrometer MALDI-TOF system and analyzed for protein mass patterns identification within a few minutes (Fig. 1).

### Statistical Analysis

The identification was considered accurate, if the direct method performed by MALDI-TOF system provided the same results as the classical method using VITEK_2_ system. The identification was considered incorrect if MALDI-TOF System provided different results from the VITEK_2_ system.

Isolates were tagged as “no identification” if the MALDI-TOF system did not provide identification results. The warning messages “bad spectrum” or “not enough peaks” appeared in the case of poor-quality deposit.

Chi square test or Fisher’s exact test was used for statistical comparisons. A *P* value <0.05 was considered statistically significant.

## Results

Out of 70 blood samples included in this study, 28 were collected and inoculated in BD BACTEC™ Plus Aerobic/F culture vials and incubated in the automated system BD BACTEC™ FX, whereas 42 samples were collected and inoculated in Oxoid Signal Blood Culture System Medium (OXOID S.p.A.).

Using MALDI-TOF system with LCM (with and without the addition of 70 % formic acid in the spots of the target plate) or VITEK_2_ system, concordance in results was obtained for in 64/70 (91.4 %) positive monomicrobial culture bottles, either for genus or species level (score values higher than 2.0 for Bruker Biotyper software).

Incorrect results were obtained for the remaining six blood culture positive bottles. In fact, 2/70 (2.9 %) isolates were identified only at the genus level system (score values between 2.0 and 1.7 for Bruker Biotyper software) and 3/70 (4.3 %) isolates were not-identified with the MALDI-TOF (not-identified for score values lower than 1.5 for Bruker Biotyper software). Misidentification occurred only for one sample (1.4 %) (Table 1).

**Table 1:** Performance of microbial identification by LCM/MALDI-TOF versus classical ID/VITEK_2_

Comparison of identification between MALDI-TOF and VITEK_2_	
Agreement	64/70 (91.4 %)
Identification only at the genus level	2/70 (2.9 %)
No identification	3/70 (4.3 %)
Misidentification	1/70 (1.4 %)

According to the type of blood culture positive bottles, results are summarized in Tables 2 and 3.

**Table 2:** Microbial identification by LCM/MALDI-TOF versus classical ID/VITEK_2_ from OXOID SIGNAL BLOOD bottles

Microorganism	Number of isolates analyzed	Correct id VITEK_2_	Correct id MALDI-TOF^a^	No id MALDI-TOF^b^	Id genus^c^	Misidentified
Gram positive	16	16	12	2	1	1
*Enterococcus faecalis*	4	4	3	1	–	–
*Enterococcus faecium*	2	2	2	–	–	–
*Staphylococcus epidermidis*	3	3	3	–	–	–
*Staphylococcus aureus*	3	3	3	–	–	–
*Streptococcus pyogenes*	1	1	1	–	–	–
*Streptococcus mitis*	1	1	–	1	–	–
*Streptococcus mutans*	1	1	–	–	–	1^d^
*Streptococcus parasanguinis*	1	1	–	–	1	–
Gram negative	25	25	25	–	–	–
*Escherichia coli*	7	7	7	–	–	–
*Klebsiella pneumoniae*	9	9	9	–	–	–
*Achromobacter xylosoxidans*	1	1	1	–	–	–
*Proteus mirabilis*	1	1	1	–	–	–
*Pseudomonas aeruginosa*	4	4	4	–	–	–
*Acinetobacter lwoffii*	1	1	1	–	–	–
*Klebsiella oxytoca*	1	1	1	–	–	–
*Enterobacter cloacae*	1	1	1	–	–	–
Yeasts	1	1	1	–	–	–
*Candida tropicalis*	1	1	1	–	–	–
Total	42					

^a^Score values higher than 2.0 for Bruker Biotyper software

^b^Score values lower than 1.5 for Bruker Biotyper software

^c^Score values between 2.0 and 1.7 for Bruker Biotyper software

^d^ *S. mutans* was misidentified as *L. plantarum*

**Table 3:** Microbial identification by LCM/MALDI-TOF versus classical ID/VITEK_2_ from BD BACTEC bottles

Microorganism	Number of isolates analyzed	Correct id VITEK_2_	Correct id MALDI-TOF^a^	No id MALDI-TOF^b^	Id genus^c^	Misidentified
Gram positive	18	18	16	1	1	–
*Enterococcus faecalis*	1	1	1	–	–	–
*Corynebacterium striatum*	1	1	–	1	–	–
*Staphylococcus epidermidis*	2	2	2	–	–	–
*Staphylococcus aureus*	8	8	8	–	–	–
*Staphylococcus capitis*	3	3	2	–	1	–
*Staphylococcus haemolyticus*	1	1	1	–	–	–
*Staphylococcus hominis*	1	1	1	–	–	–
*Streptococcus salivarius*	1	1	1	–	–	–
Gram negative	9	9	9	–	–	–
*Escherichia colii*	1	1	1	–	–	–
*Klebsiella pneumoniae*	4	4	4	–	–	–
*Serratia marcescens*	1	1	1	–	–	–
*Stenotrophomonas maltophilia*	1	1	1	–	–	–
*Pseudomonas aeruginosa*	2	2	2	–	–	–
Yeasts	1	1	1	–	–	–
*Candida albicans*	1	1	1	–	–	–
Total	28					

^a^Score values higher than 2.0 for Bruker Biotyper software

^b^Score values lower than 1.5 for Bruker Biotyper software

^c^Score values between 2.0 and 1.7 for Bruker Biotyper software

Table 2 shows identification results of LCM/MALDI-TOF versus classical ID/VITEK_2_ from OXOID SIGNAL BLOOD bottles. Out of 42 samples, 16 g-positive were isolated and only 12 were correctly identified by MALDI-TOF system with LCM with and without the addition of 70 % formic acid in the spots of the target plate (score values higher than 2.0 for Bruker Biotyper software).

Two samples were scored as “not-identified”: in fact the MALDI-TOF system did not reveal the microbial species either for at genus or species level (*Enterococcus faecalis and Streptococcus mitis*) (not-identified for score values lower than 1.5 for Bruker Biotyper software) (Table 2).

In one case, the MALDI-TOF system revealed the microbial species at the genus level but not at the species level (*Streptococcus parasanguinis* was identified as *Streptococcus mitis/oralis*) (score values between 2.0 and 1.7 for Bruker Biotyper software).

A misidentification occurred for only one sample: *Streptococcus mutans* was misidentified as *Lactobacillus plantarum* (Table 2).

About the identification of gram-negative microorganisms, 25 isolates were correctly recognized with a perfect agreement by both utilized systems VITEK_2_ and MALDI-TOF (score values higher than 2.0 for Bruker Biotyper software) (Table 2).

Moreover, the LCM with the addition of 70 % formic acid, has also allowed us to identify microorganisms belonging to the class of yeasts. In fact, in one sample a *Candida tropicalis* was revealed by both analytical systems (score values higher than 2.0 for Bruker Biotyper software) (Table 2).

In Table 3, identification results by LCM/MALDI-TOF vs classical ID/VITEK_2_ from BD BACTEC bottles are shown. Out of 28 samples, 18 g-positive were isolated and only 16 were correctly identified by MALDI-TOF system (score values higher than 2.0 for Bruker Biotyper software).

Incorrect results were obtained for the remaining two blood culture positive bottles. In one sample MALDI-TOF failed to give the identification of species (*Staphylococcus epidermidis* was identified as *Staphylococcus capitis*) (score values between 2.0 and 1.7 for Bruker Biotyper software), whereas the other sample was scored as “not-identified”: in fact the MALDI-TOF system did not reveal the microbial species either for genus or species level (*Corynebacterium striatum*) (not-identified for score values lower than 1.5 for Bruker Biotyper software) (Table 3).

About the identification of gram-negative microorganisms, there was a perfect correspondence, since all the blood culture positive bottles were identified successfully by both VITEK_2_ and MALDI-TOF system (score values higher than 2.0 for Bruker Biotyper software) (Table 3).

Also in this case, the LCM with the addition of 70 % formic acid allowed us to correctly identify microorganisms belonging to the class of yeasts: *Candida albicans* was revealed by both analytical systems (score values higher than 2.0 for Bruker Biotyper software) (Table 3).

Finally, in Table 4 the identification rate performed using MALDI-TOF system is reported, according to the type of positive blood culture bottles. The correct identification rate by LCM direct method was 38/42 (90.4 %) and 26/28 (93 %) for OXOID SIGNAL BLOOD and BD BACTEC bottles, respectively (*P* = 0.916). No identification was observed in 2/42 (4.8 %) OXOID SIGNAL BLOOD bottles and in 1/28 (3.5 %) BD BACTEC bottles (*P* = 0.710).

**Table 4:** Identification performed using MALDI-TOF system according to the type of positive blood culture bottles

	Oxoid signal blood	Bactec	*P* value
Patients (*N* = 70)			
Correct identification	90.4 % (38/42)	93 % (26/28)	0.916
No identification	4.8 % (2/42)	3.5 % (1/28)	0.710
Misidentification	2.4 % (1/42)	–	0.828
Genus identification (no species)	2.4 % (1/42)	3.5 % (1/28)	0.654

Identification results between OXOID SIGNAL BLOOD bottles and BD BACTEC bottles were not statistically different

A misidentification occurred in 1/42 (2.4 %) OXOID SIGNAL BLOOD bottles and in 0/28 BD BACTEC bottles (*P* = 0.828). Finally, a genus identification was found in 1/42 (2.4 %) OXOID SIGNAL BLOOD bottles and in 1/28 BD BACTEC bottles (*P* = 0.654).

## Discussion

BSI are a significant source of mortality and morbidity. Patient outcomes are improved by rapid identification of the causative pathogen and administration of appropriate antimicrobial therapy [1, 18].

Until recently, microbial identification in clinical diagnostic laboratories has mainly relied on conventional phenotypic and gene sequencing identification techniques. The development of MALDI-TOF MS has updated the routine identification of microorganisms in clinical microbiology laboratories by introducing an easy, rapid, low-cost, and efficient identification technique [7].

In the present study we probed a more simple, fast, and reliable protocol for correct identification of monomicrobial infection in the blood, using a new recovery method for microorganisms from positive blood culture bottles. The LCM for MALDI-TOF System was compared with the routine method, in order to overcome the limitations, mainly related to the long times required for infectious signaling and identification, using conventional systems as the VITEK_2_.

The application of LCM before the identification by the MALDI-TOF system [5], has allowed to correctly identify a 91.4 % of microorganisms responsible for bacteremia with an agreement to the species and the genus level (score values higher than 2.0 for Bruker Biotyper software), whereas only in the remaining 8.6 % the MALDI-TOF system has not produced a reliable identification.

When compared with the standard method VITEK_2_, the direct method showed rapid and reliable results, especially for the gram-negative group, in accordance with the results of other studies [8, 10].

Regarding the gram-positive identification, our results have evidenced a relatively low correct identification rate by LCM (with and without the addition of 70 % formic acid in the spots of the target plate) before MALDI-TOF in comparison to conventional method. In fact, as reported in Tables 2 and 3, 12/16 g-positive obtained from 42 samples in OXOID SIGNAL BLOOD and 16/18 g-positive obtained from 28 BD BACTEC bottles were correctly identified by MALDI-TOF system, respectively. In particular, the MALDI-TOF system (i) did not reveal the microbial species either for genus and species level or (ii) revealed the microbial species at the genus level but not at the species level.

These data coincide with the previous data existing in literature. As observed by others and noted in the limitations of MALDI-TOF reported by the manufacturer, the lower yield of valid MALDI-TOF results with streptococci and staphylococci might be due to the difficulty to distinguish between members of closely related group members of streptococci belonging to the *S. mitis* group (i.e., *S. pneumoniae, S. mitis, S. sanguinis, S. oralis*), that did not produce very high scores [11, 13, 17, 19–21, 25].

Moreover, the cell wall composition of gram-positive bacteria conferring increased resistance to lysis and the possible presence of some residual blood proteins could be considered a limit for a correct identification by MALDI-TOF [20]. In fact, some authors, for a better disruption of the peptidoglycan layer of the gram-positive bacterial cell walls, proposed a treatment with ultrasound [10].

However, our data show a better performance on the use of MALDI-TOF directly from positive blood cultures by LCM with respect to Sepsityper system used by other authors (91.4 vs 85.2 and 85.5 %) [13].

Moreover as reported by Kok et al. [11] the rapid turnaround time of MALDI-TOF MS in the direct identification of blood culture pathogens is more useful for gram-negative bacteremias, where knowledge of the bacterial pathogen can have a more significant impact on the choice of antibiotics because of their antimicrobial resistance mechanisms.

Finally concerning the yeasts identification, despite the paucity of positive samples for yeast, our results correctly identify *C. tropicalis* and *C. albicans* by both analytical systems.

Excluding the “no identification” and the “misidentification” results, the concordance rate of identification between the direct method and the standard method was very reliable [38/42 (90.4 %) and 26/28 (93 %)] for OXOID SIGNAL BLOOD and BD BACTEC bottles, respectively (*P* = 0.916).

In conclusion, our simple and cost-effective sample preparation method would be very useful for rapid identification of microorganisms from positive blood culture bottles. The rapid turnaround time of 2 h for direct LCM compares favorably with the timeframe required for identification by conventional methods (24/48 h) after the initial “signaling” of organisms in blood cultures.

MALDI-TOF MS is a rapid and accurate method for the identification of pathogens from positive blood culture broths. Variations in methodology of blood culture broth processing should be taken into consideration when interpreting results from this and other studies. The identification of gram-positive bacteria should improve with ongoing technical development and further refinement of the reference spectra within the MALDI Biotyper database. We are just studying a new technical development to overcome the gram-positive misidentification and reach valid results as the gram-negative’s one.

In future, the identification of the etiologic pathogen in bacteremic patients presenting with shock within the critical 6 h will further reduce morbidity and mortality.
